# Desmoid Tumors—Experience from a Referral Center, Part 1: Multidisciplinary Review and Practical Recommendations

**DOI:** 10.3390/cancers17213470

**Published:** 2025-10-29

**Authors:** Rosa Alvarez Alvarez, Carolina Agra Pujol, Marta Arregui Valles, Francisco Alijo, Adriana Fernández Gonzalo, Natalia Gutiérrez, Pablo Lozano Lominchar, Cristina Mata Fernández, Lidia Mediavilla Santos, Ulrike Novo, Marina Santos, Guillermo Hernández Torrado, Henar Carpintero García, Ana Gutiérrez-Ortiz de la Tabla

**Affiliations:** 1 Medical Oncology Department, Instituto de Investigacion Sanitaria Gregorio Marañon, Hospital General Universitario Gregorio Marañon, 28007 Madrid, Spain; marta.arregui@salud.madrid.org (M.A.V.); ngutierrezalonso@salud.madrid.org (N.G.); ghernandezt@salud.madrid.org (G.H.T.); 2 Department of Pathology, Hospital Gregorio Marañon, Complutense University, 28007 Madrid, Spain; carolina.agra@salud.madrid.org; 3 Radiology Department, Hospital General Universitario Gregorio Marañon, 28007 Madrid, Spain; francisco.alijo@salud.madrid.org (F.A.); afgonzalo@salud.madrid.org (A.F.G.); ulrike.novo@salud.madrid.org (U.N.); 4 General Surgery and Surgical Oncology Department, Hospital Universitario Gregorio Marañon, Complutense University, 28007 Madrid, Spain; pablo.lozano@salud.madrid.org; 5 Pediatric and Adolescent Oncohematology Unit, Pediatric Sarcomas National Reference Center, Hospital General Universitario Gregorio Marañón, 28007 Madrid, Spain; cristina.mata@salud.madrid.org; 6 Orthopedic Surgery and Traumatology Department, Musculoskeletal Oncology Section, Hospital Universitario Gregorio Marañon, 28007 Madrid, Spain; lydia.mediavilla@salud.madrid.org (L.M.S.); henar.carpintero@gmail.com (H.C.G.); 7 Radiation Oncology Department, Hospital Universitario Gregorio Marañon, 28007 Madrid, Spain; marina.santos@salud.madrid.org; 8 Medical Oncology Department, Hospital Universitario Infanta Leonor, 28031 Madrid, Spain; agutierrezo@salud.madrid.org

**Keywords:** desmoid-type fibromatosis, intra-abdominal desmoids, extra-abdominal desmoids, familial adenomatous polyposis, CTNNB1 mutation, active surveillance, multidisciplinary management, referral center experience, desmoid tumor

## Abstract

**Simple Summary:**

Desmoid tumors are rare soft tissue neoplasms, with an estimated incidence of 2 to 4 cases per million inhabitants per year and highly variable clinical behavior. Their low frequency and heterogeneous presentation make diagnosis and treatment particularly difficult, requiring evaluation and treatment at specialized referral centers. This study summarizes the recommendations for the diagnosis and treatment of desmoid tumors at our national referral center and reviews the most recent therapeutic advances developed for this disease. This collaborative work by a multidisciplinary team highlights the importance of specialized, coordinated, and patient-centered care to ensure optimal clinical outcomes in this uncommon disease.

**Abstract:**

Desmoid tumors (DTs), also known as aggressive fibromatosis, are rare neoplasms characterized by local invasiveness and a high risk of recurrence, despite their lack of metastatic potential. The management of these tumors remains challenging due to their unpredictable behavior and heterogeneous presentations. In this two-part study, we first provide a comprehensive review of the scientific evidence on diagnosis and emerging therapeutic strategies for DT. In the second part, we will present a retrospective analysis of our experience at a national reference center for sarcoma treatment, focusing on diagnostic strategies, therapeutic interventions, and clinical outcomes.

## 1. Introduction

Desmoid tumors represent a rare clinical entity characterized by a monoclonal proliferation of fibroblasts [1]. According to the World Health Organization (WHO) classification of soft-tissue and bone tumors, these entities are categorized as intermediate tumors due to their locally aggressive behavior and simultaneous lack of capacity to metastasize [2]. The incidence of DT is approximately 2–4 cases per million per year, accounting for less than 3% of all soft tissue sarcomas. The median age at diagnosis is between 30 and 40 years, with a higher prevalence among females (female-to-male ratio of 3:2).

The most common anatomical locations for sporadic cases are the trunk and extremities, whereas intra-abdominal, abdominal wall, and multifocal tumors are frequently observed in patients with FAP [3].

While the majority of DTs arise sporadically, approximately 10–15% may be associated with familial adenomatous polyposis (FAP), involving mutations in the *Adenomatous Polyposis Coli* (APC) gene. In both sporadic and FAP-related cases, there is a dysregulation of the Wnt/beta-catenin pathway, resulting in impaired degradation of beta-catenin and its subsequent accumulation, promoting cellular proliferation. Mutations in *CTNNB1* (beta-catenin encoding gene) are observed in up to 80% of sporadic DTs [4].

Several risk factors have been identified, including a history of trauma, which is particularly relevant for FAP, patients with up to a 50% risk of tumor development at surgical sites [5].

Pregnancy is also correlated with DT, potentially due to estrogen receptor expression [6]. The likelihood of tumor progression during pregnancy is high; however, pregnancy does not seem to adversely affect the outcomes of subsequent treatments. Notably, as many as 30% of patients do not require any intervention [7]. Furthermore, pregnancy does not appear to increase the risk of recurrence following the resection of a DT [8].

Clinically, DT often presents as a slow-growing mass associated with pain or as intra-abdominal lesions causing non-specific digestive complaints, potentially leading to bowel obstruction.

The natural history of DT is highly heterogeneous, ranging from aggressive growth to spontaneous regression. Extra-abdominal DTs have low mortality rates, generally less than 1%. However, this rate increases to approximately 10% for intra-abdominal tumors, particularly in patients with FAP [9].

Follow-up is usually conducted using computed tomography (CT) scans for intra-abdominal tumors and magnetic resonance imaging (MRI) for tumors located in the extremities or trunk. The frequency of these imaging tests should be tailored to the tumor’s clinical behavior and location, with recommendations for evaluations every 2–6 months during the initial follow-up period [10].

Colonoscopy at the time of diagnosis is advised, especially for intra-abdominal cases, to exclude the presence of colonic polyps. Additionally, genetic testing is recommended to rule out FAP, particularly in patients presenting with intra-abdominal or multifocal tumors, those under 40 years of age, or those with a family history of colonic polyps [11].

## 2. Methodology

These recommendations were developed by a multidisciplinary team of specialists from various fields involved in the diagnosis and management of DTs at a referral center. A literature review was conducted using PubMed, and international guidelines—including those from the NCCN, ESMO, and EURACAN—were consulted, along with relevant abstracts presented at international meetings. During a consensus meeting, each section was presented by an expert to the group for discussion. The coordinating author (RA) was responsible for compiling and standardizing the content of the different sections. All authors reviewed and approved the final version of the document. The panel adopted the levels of evidence (I to V) and grades of recommendation (A to C) established by the Infectious Disease Society of America [12], as shown in Table 1.

## 3. Diagnostic Approach to DT

### 3.1. Imaging Diagnosis

The main role of radiological examination is not only to establish the diagnosis of DT but also to define its extension and potential image-guided cryoablation treatment or resectability. It is also essential in the follow-up of tumors managed with active surveillance (AS), the initial treatment strategy for most DTs [13].

Ultrasonography (US) is the first-line imaging technique used to evaluate palpable lesions and as a guide for biopsy. The sonographic appearance is variable, usually as oval masses with lobulated or poorly defined margins that may show vascularity in Doppler studies [14]. Due to the heterogeneous composition, these lesions may appear hypoechoic if matrix and collagen prevail, or hyperechoic if there is more cellular stroma.

CT and MRI are the modalities of choice for image-guided treatment and surgical planning. CT may show an ill-defined mass with infiltrative margins or a homogeneous appearance with well-defined borders, iso- or slightly hyperdense relative to muscle. This technique is preferred in the follow-up of patients with intra-abdominal DT to evaluate potential complications during the disease, as in local aggressive cases, they may invade contiguous structures and cause small-bowel obstruction or hydronephrosis [15].

MRI provides better soft tissue contrast resolution, making it the preferred modality to evaluate extra-abdominal desmoids and to assess the relationship between the tumor and surrounding neurovascular structures [16].

**Figure 1:**
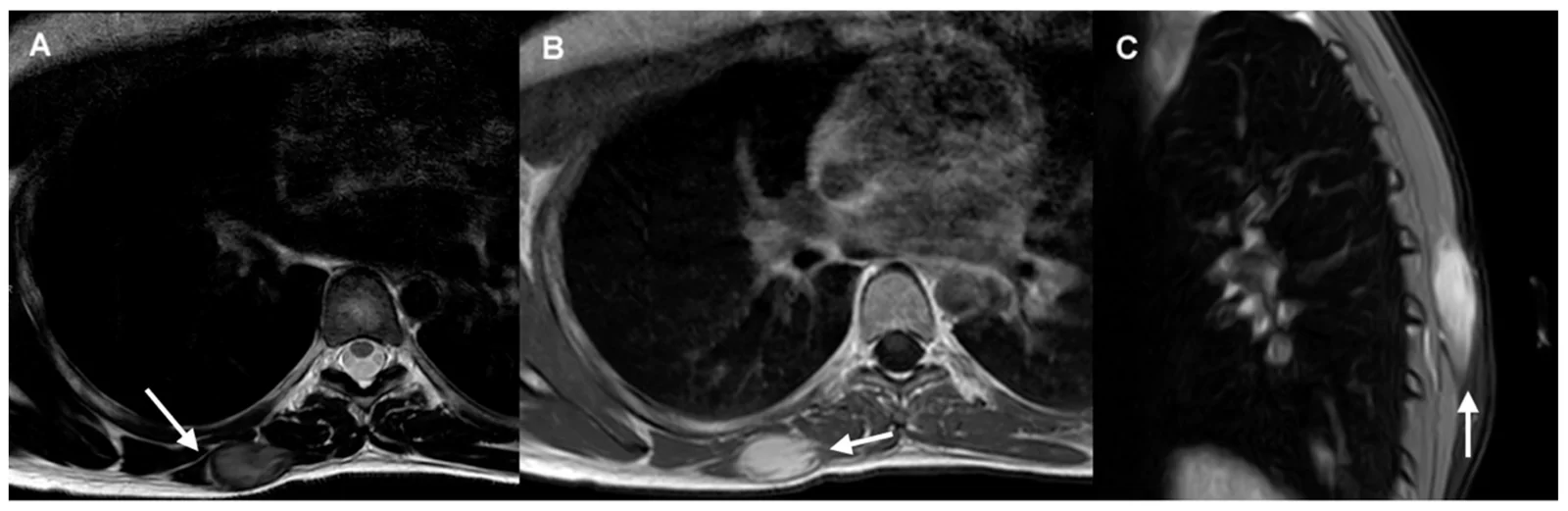
DT of the right distal trapezius muscle (Scale 11.9×). (**A**) The axial T2-weighted MRI sequence demonstrates a well-defined mass with intermediate signal and hypointense internal collagen bundles. The “split-fat sign” is shown in its lateral margin (arrow). (**B**) Axial post-contrast T1-weighted MRI sequence shows homogeneous mass enhancement and “staghorn sign” (arrow). (**C**) Sagittal fat-suppressed image shows “fascial tail sign” (arrow).

Certain imaging features represent local invasion, such as the “staghorn sign”, which corresponds to intramuscular finger-like extensions, and “fascial tail sign”, an infiltrative border due to linear extension along fascial planes [12]. MRI may also demonstrate the “split fat sign”, which consists of a halo of fat surrounding the tumor (Figure 1).

MRI signal intensity is the result of the proportion of spindle cells, collagen fibers, and extracellular matrix [13]. On T2-weighted images, signal intensity is usually intermediate, frequently with internal hypointense bands corresponding to collagen bundles. As the disease evolves, cellularity decreases while collagen deposition and fibrosis increase, resulting in hypointense lesions on T1 and T2-weighted sequences [17]. Recurrent or actively growing desmoids usually have a higher T2 signal and contrast enhancement caused by hypercellularity [18,19], as shown in Figure 1.

Recommendations:

- US is recommended as the first-line imaging modality for the initial evaluation of palpable lesions and for guiding core needle biopsy in DTs (IV, B).
- CT and MRI are the imaging modalities of choice for treatment planning, image-guided procedures, and follow-up in patients with DTs (IV, B).
- MRI is considered the optimal imaging modality for evaluating extra-abdominal DTs (IV, B).
- CT is preferred in the follow-up of intra-abdominal DTs, particularly for evaluating the extent of disease and identifying potential complications (IV, B).

### 3.2. Biopsy

Histopathological diagnosis is crucial in developing effective treatment strategies for DT. Core needle biopsy, performed under US or CT guidance, is minimally invasive and offers not only high diagnostic accuracy but also the opportunity for molecular analyses, including the detection of CTNNB1 mutations, which are key drivers in DT pathogenesis and may influence treatment decisions [20].

Among the techniques used for biopsy, ultrasound-guided core needle biopsy is particularly advantageous for soft tissue tumors such as DT. This method provides real-time imaging, enhancing both safety and accuracy. Typically, 18G or 16G needles are used to obtain sufficient material for both histological and molecular evaluation [21].

- Recommendation: Core needle biopsy is the standard method for the diagnosis of DTs (IV, A)

### 3.3. Histological Diagnosis

**Figure 2:**
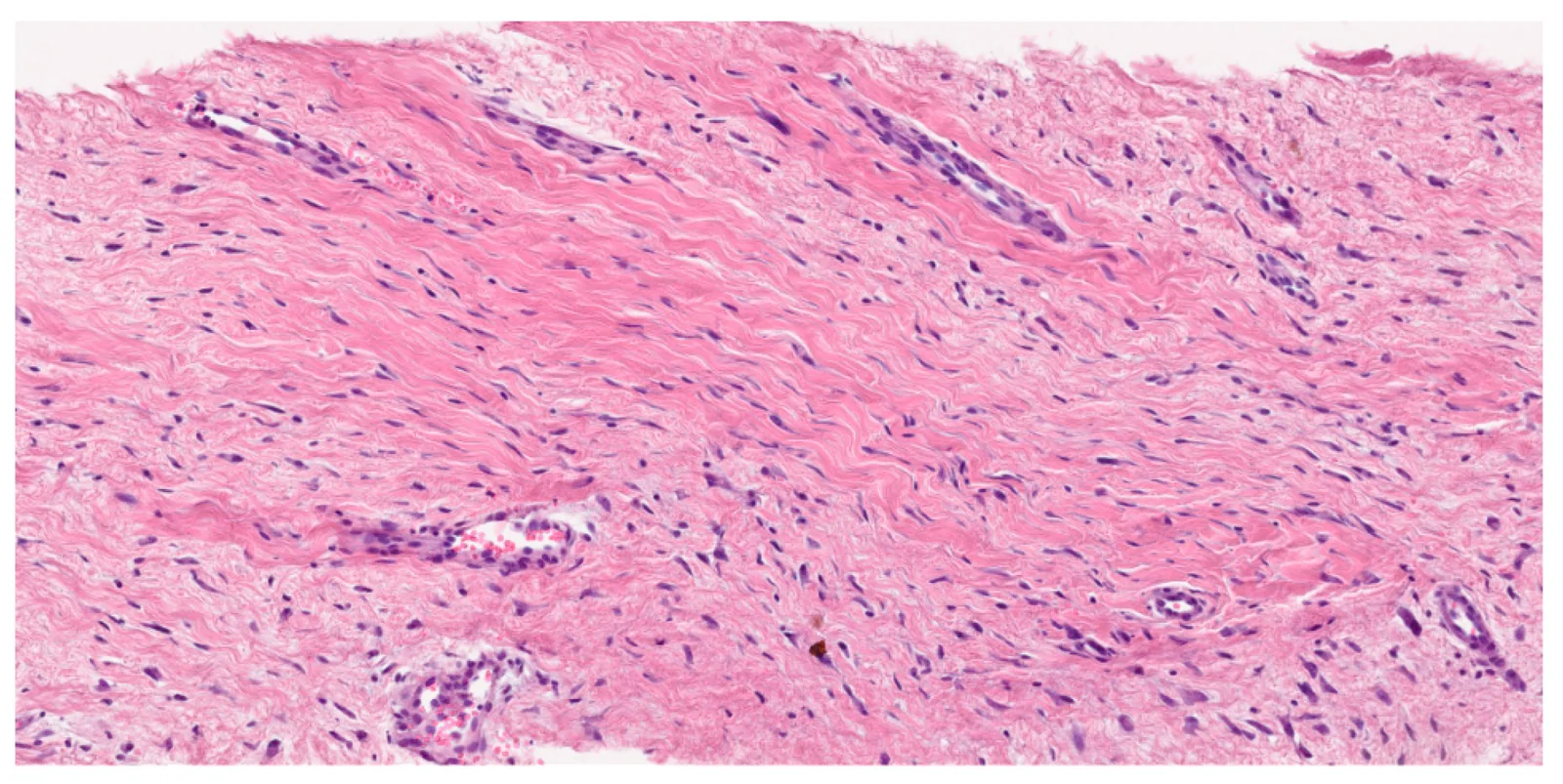
Hematoxilin–Eosine (scale 16.6×). Long, sweeping fascicles of bland fibroblasts without atypia or hyperchromasia.

Desmoid fibromatosis is a low-grade, isomorphic spindle cell neoplasm composed of bland fibroblasts and myofibroblasts with infiltrative borders, arranged in long, sweeping fascicles. Characteristically, it features a collagenous stroma (containing rounded, intermediate-caliber vessels with subtle perivascular edema). The uniform lesional cells display a pale eosinophilic cytoplasm, tapering nuclei, fine chromatin (with no nuclear hyperchromasia or atypia), and rare or absent mitotic activity. Atypical mitosis is not observed. In addition to this conventional pattern, some examples can be hyalinized/hypocellular, myxoid, keloidal (a finding most common in intra-abdominal tumors), nodular fasciitis-like (especially when arising in mesentery), and hypercellular, sometimes with staghorn vessels (Solitary Fibrous Tumor-like vessels). A case with nuclear pleomorphism and TP53 mutation has been described by Foster and coworkers [22]. Please see Figure 2.

According to immunohistochemistry (IHC), the cells are positive for SMA and MSA and aberrant nuclear beta-catenin expression (80% of sporadic desmoid fibromatosis and 67% for patients with FAP, and 56% of superficial fibromatosis). In the correct morphologic context, nuclear beta-catenin expression would support the diagnosis of DT.

The nuclear expression of beta-catenin immunostaining is equivocal or challenging to interpret (most cases show cytoplasmic positivity, which is nonspecific and can occur in other tumors). Therefore, betacatenin negativity does not preclude the diagnosis of desmoid fibromatosis.

In addition, a variety of spindle cell neoplasms are nuclear-positive for beta-catenin: low-grade myofibroblastic sarcoma (LGMS), gastrointestinal stromal tumor (GIST), solitary fibrous tumor (SFT), and desmoplastic fibroblastoma (DF). Nevertheless, this distinction may be difficult when using needle biopsy.

It is advisable to perform an immunohistochemical panel to aid in differential diagnosis in the context of other tumors (depending on the location), and particularly of scar tissue, which represents the main differential diagnosis [23]. The histological differential diagnosis of DTs involves the careful evaluation of cellular architecture, the arrangement of collagen fibers, vasculature, cell pleomorphism, and the presence of specific features (nerve fibers, inflammatory cells, or histiocytes to rule out scarring) [24].

**Figure 3:**
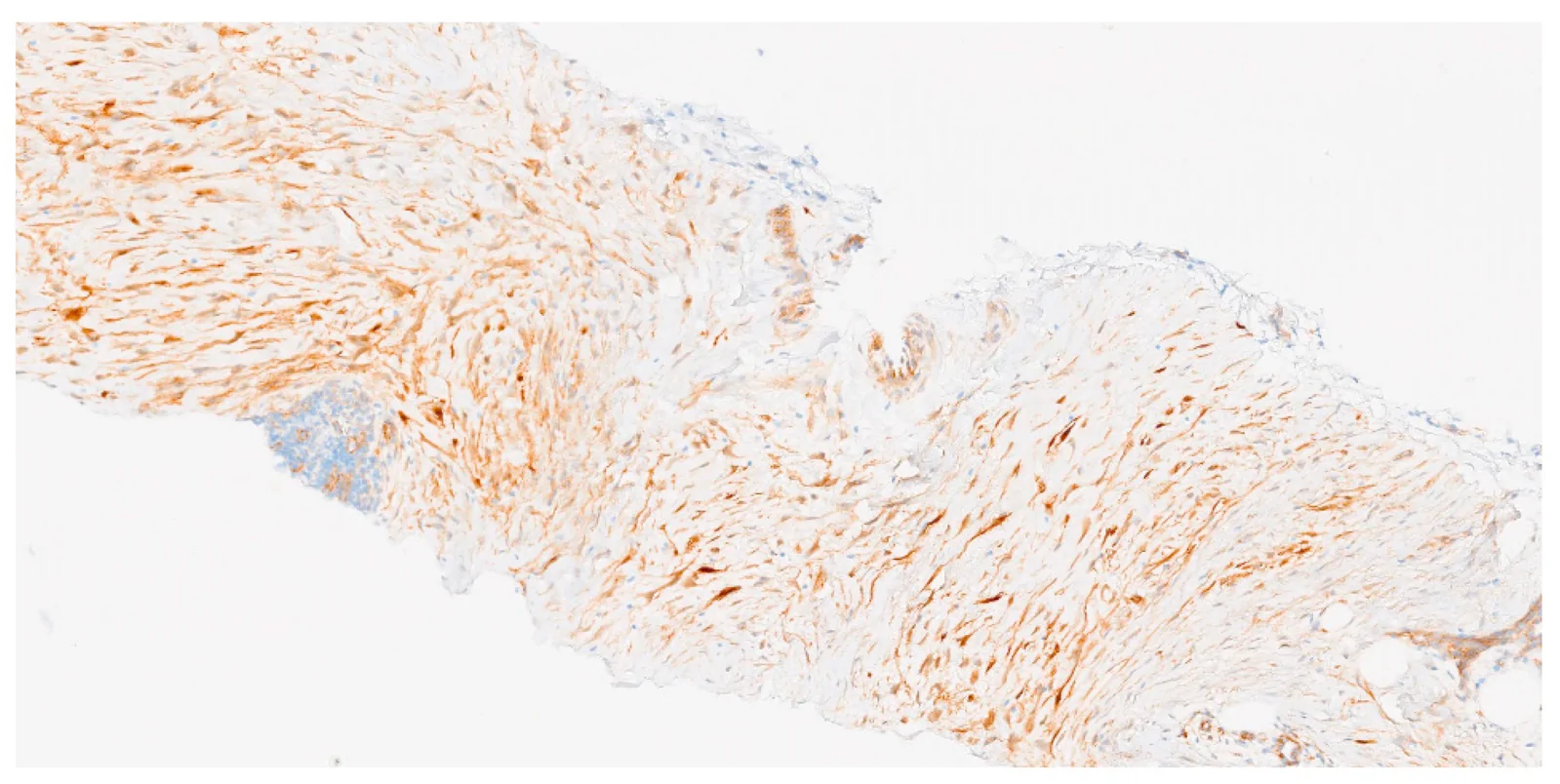
Beta-catenin immunochemistry (scale 11.9×). Beta-catenin expression with focal and intense nuclear reactivity.

CTNNB1 mutation analysis may be helpful in small biopsy specimens when diagnostic morphological features are not readily apparent. CTTNB1 mutations and APC mutations are mutually exclusive in DT. Please see Figure 3.

### 3.4. Indication for Molecular Study

Genetic causes in DT are predominantly somatic, activating mutations of the beta-catenin gene (CTNNB1 exon 3), which encodes the beta-catenin protein, or the loss of APC. These mutations are mutually exclusive. Thus, the detection of a somatic CTNNB1 mutation can help to exclude a syndromic condition, or vice versa. CTNNB1 wild-type status (especially in intra-abdominal cases) can be ruled out by FAP with more clinical studies and mutational analyses.

Several factors support the use of molecular testing in DTs:

1. Diagnostic confirmation. Although most desmoid tumors are diagnosed based on histological and clinical features, molecular analysis can aid in confirming the differential diagnosis in complex or doubtful cases when beta-catenin immunostaining is equivocal [2];
2. Exclusion of syndromic disease. In patients with a personal or family history of FAP, the analysis of the *APC gene* is critical. Notably, 85–90% of desmoid tumors associated with FAP harbor APC mutations;
3. Assessment of CTNNB1 mutation status and recurrence risk. Certain CTNNB1 mutations, particularly p.Ser45Phe, have been associated with a higher risk of local recurrence. Determining the mutation status may therefore provide valuable prognostic information and guide clinical follow-up and treatment planning;
4. Identification of potential therapeutic targets. In selected cases, molecular profiling may help identify therapeutically actionable targets, such as alterations in the Wnt/β-catenin pathway, which may be amenable to targeted therapies, especially in sporadic DTs with CTNNB1 mutations;
5. Detection of additional relevant genetic alterations. Beyond CTNNB1 and APC, other genetic changes—including chromosome 22 rearrangements and mutations in genes related to cell proliferation and tumor invasion—may be identified. These findings can contribute to a deeper understanding of DT biology and the mechanisms underlying tumor development.

Recommendation

- Pathological diagnosis should be made by a sarcoma expert pathologist according to the most current WHO classification of soft tissue and bone tumors (IV, A).
- Molecular testing is recommended to confirm the diagnosis in histologically or immunohistochemically equivocal cases and to exclude syndromic conditions (FAP) in patients with CTNNB1 wild-type DTs (IV, B).
- Genotyping for specific CTNNB1 mutations (S45F) may be considered to estimate the risk of local recurrence and guide surveillance intensity (IV, C).
- Molecular profiling may be useful in selected cases to identify potential therapeutic targets (V, C).

## 4. Management of DT

### 4.1. Surgical Approach

Historically, surgery has been the first-line treatment in DT. However, the high rates of local recurrence and significant surgical morbidity have shifted the paradigm toward an initial AS strategy. Current guidelines advocate for a conservative approach in the management of DTs, reserving surgery for carefully selected cases [1,25].

#### 4.1.1. Intra-Abdominal DT

When addressing the complex scenario of intra-abdominal desmoid tumors, the surgical approach within a multidisciplinary team at a sarcoma referral center should be based on three key pillars: understanding molecular biology, determining indications for active treatment, and evaluating systemic treatment options considering response rates, response speed, and associated side effects. Current expert consensus applies the same treatment algorithm to all desmoid tumors, regardless of location [25]. Although the GRAFITI trial did not include patients with intra-abdominal desmoids, it supports an AS strategy, with active treatment considered only in cases of radiological or symptomatic progression [26]. However, the intra-abdominal location presents distinct challenges when comparing FAP-related DTs versus sporadic cases. A mesenteric root desmoid is not equivalent to a 4 cm lesion in the distal ileum mesentery, nor are segmental resection or right hemicolectomy comparable to total enterectomy or pelvic exenteration. The feasibility of monitoring intra-abdominal DTs and defining significant symptoms remains a critical consideration, highlighting the complexity of these tumors for both surgeons and multidisciplinary teams. Tumor biology in intra-abdominal desmoid-type fibromatosis encompasses factors such as initial tumor size, obstructive symptoms, perforation, fistulization, and growth rate. The prognostic significance of molecular markers remains unclear. While the GRAFITI trial indicated that patients with S45F mutations had a higher likelihood of requiring active treatment initiation, this finding has not yet dictated treatment decisions. However, *APC* gene mutations in patients with FAP are clinically significant, as these tumors exhibit high recurrence rates and frequent multifocality, necessitating a distinct management approach [27,28].

For intra-abdominal DTs not associated with FAP, recent consensus guidelines support surgery when the associated morbidity is not prohibitive. Surgery should be avoided in cases where morbidity could result in short bowel syndrome or total pelvic exenteration. Because the risk of local recurrence increases significantly after an R1 resection, performing an R0 resection is the goal. Function- and organ-preserving surgery should be prioritized whenever possible. There is no indication to extend margins in R1 resections, as confirmed by a meta-analysis assessing the impact of surgical margins and postoperative radiotherapy (RT) on local recurrence in sporadic desmoid-type fibromatosis [29]. This study, which included 16 reports and 1295 patients, found that the local recurrence risk was nearly twice as high for patients with positive microscopic margins (RR 1.78, 95% CI 1.40–2.26). Additionally, postoperative RT following negative-margin surgery provided no detectable recurrence benefit, whereas it improved outcomes after incomplete resection in both primary tumors (RR 1.54, 1.05–2.27) and recurrent DF (RR 1.60, 1.12–2.28).

In FAP-associated DT, surgery should be avoided whenever possible, including biopsy procedures. Emerging data suggest a potential role for intestinal transplantation, with ongoing investigations. Data from the Roy Calne Transplant Unit in Cambridge, United Kingdom, indicate that between October 2007 and September 2023, 144 intestinal transplants (ITx) were performed in 130 patients, including 15 (9%) for FAP-associated desmoid. Five patients underwent a sequential procedure, while 10 underwent simultaneous resection and ITx. The 5-year patient survival rate was 82%, with 10 of 15 patients (67%) alive at the time of reporting [30].

The 2024 consensus has reinforced surgical indications for progressive intra-abdominal DTs, but a careful assessment of procedural morbidity remains essential [25]. Alternative management strategies, including systemic medical treatment, should be considered based on patient-specific factors.

Recommendations

- Surgery is indicated in cases of severe intra-abdominal complications in sporadic DTs (IV, B).
- In FAP-associated DTs, surgery should be avoided whenever possible and only considered in life-threatening situations. Surgical intervention may be warranted in cases of tumor progression if AS fails (IV, B).

#### 4.1.2. Abdominal Wall DT

The abdominal wall is the most common site of sporadic desmoid tumors. When active treatment is indicated, surgery is considered a first-line option, comparable to systemic therapies and local ablative approaches. Wide negative margins are desirable when feasible; however, narrow or microscopically positive margins do not necessarily correlate with higher recurrence risk, given the unpredictable biological behavior of desmoid tumors. Surgical resection is associated with a low local recurrence rate—approximately 5%—and minimal morbidity. Wilkinson et al. reported a retrospective series of 55 patients who underwent resection for primary sporadic abdominal wall fibromatosis. R0 resection was achieved in 50% of cases, and disease-free survival at 6 years was approximately 90% [31]. Several retrospective studies have evaluated outcomes following the surgical resection of abdominal wall desmoids. Across five series published between 1989 and 2013, a total of 118 patients were included, with a marked female predominance (92%). Median tumor sizes ranged from 5 to 11 cm, and follow-up periods from 4 to 8 years. Recurrence rates were consistently low, with only six cases (5.1%) reported overall. Notably, studies by Catania and Bertan reported no recurrences, while the highest rate (16%) was observed in a small cohort by Sutton [32,33,34,35]. These findings underscore the effectiveness of surgical resection in managing abdominal wall desmoid tumors. Surgical management may require extensive myofascial resection and abdominal wall reconstruction using mesh, flaps, or primary closure. The choice of technique depends on the extent of the defect and individual patient factors, such as pregnancy status.

Recommendation:

- In sporadic DTs of the abdominal wall requiring active treatment, surgery should be considered a first-line option, alongside systemic therapies and local ablative approaches (IV, B).

#### 4.1.3. Extra-Abdominal DT

In extra-abdominal DT, surgical intervention is mainly indicated in cases of severe symptoms, where there is tumor progression despite systemic therapies, or persistent/recurrent disease after non-surgical treatment [25,36]. Wide excision with negative margins is crucial for a successful surgical approach. Current recommendations suggest resecting the tumor with at least 1–2 cm of healthy surrounding tissue to minimize the risk of local recurrence. Despite meticulous surgical application, DTs maintain a high propensity for local relapse, with recurrence rates ranging between 20% and 60%, even following complete (R0) resections [30,37]. These findings underscore the infiltrative and biologically unpredictable behavior of desmoid tumors.

However, surgical management in extra-abdominal DT is associated with notable complications. Recurrence remains a significant concern, even when negative margins are achieved. Moreover, due to the necessity for wide resections, reconstructive procedures, including local or pedicled flaps, are frequently required, adding morbidity at both the recipient and donor sites [31,38]. Functional impairments, wound complications such as dehiscence or infection, neuropathic pain, and disfiguring scars are also commonly reported, with considerable impact on patients’ quality of life.

Given the substantial morbidity associated with extensive resections, surgery is increasingly considered a last resort, reserved for patients with severe symptoms or tumors threatening critical structures [1,39]. Multidisciplinary evaluation is essential, involving surgical oncologists, medical oncologists, radiologists, reconstructive surgeons, and pathologists in assessing individual cases comprehensively and optimizing outcomes.

Experience from specialized sarcoma centers suggests that, in selected patients, surgical resection can provide durable local control. Nevertheless, the decision to proceed with surgery must be highly individualized, balancing potential oncological benefits against the risks of postoperative complications and long-term functional consequences [31,32].

Recommendations:

- Surgery is indicated in cases of localized and easily resectable tumors with symptomatic disease (pain or functional impairment), especially when previous non-surgical approaches have failed (IV, B).
- Function-preserving surgery is highly recommended, prioritizing quality of life over obtaining wide resection margins (V, B).

### 4.2. Local Ablative Techniques

Cryoablation is currently one of the most effective and widely used local ablation techniques. It is a minimally invasive option that may be considered especially for patients with progressive, symptomatic, or recurrent DT [19,40]. This image-guided percutaneous technique induces tumor destruction through cycles of freezing and thawing, causing intracellular ice formation, endothelial damage, and ischemia [34]. Pre-procedural planning typically involves an MRI to evaluate tumor morphology and its relationship with critical anatomical structures [34,41].

Recent clinical trials have derived promising results with this approach. The CRYODESMO-01 study is a prospective multicenter trial that reported a disease control rate of 85–90% at 12 months. In addition, cryotherapy was associated with a significant reduction in pain and improved function [19]. Other studies describe local control rates of over 80% and lower recurrence compared to surgical removal [34,35].

Cryoablation is generally well tolerated, with minor complication rates ranging from 6% to 24%, primarily consisting of minor issues (e.g., temporary pain, edema, or neuropathy) [42]. Major complications, such as nerve injury or fracture, are rare, and can be prevented by careful planning and the use of methods such as hydrodissection [36,43]. This technique involves the injection of fluid between the tumor and adjacent critical structures, thereby creating a separation plane that minimizes the risk of thermal injury.

Other ablative techniques include radiofrequency ablation (RFA), which induces coagulative necrosis using high-frequency currents. Although beneficial in small, well-circumscribed lesions, its effectiveness can be hindered in tight collagenous tissue or neoplasms close to nerves due to thermal risk [34,44]. Microwave ablation (MWA) generates larger, more uniform ablation zones and is less tissue-impedance-sensitive but may increase the risk of collateral damage [34]. In parallel, high-intensity focused ultrasound (HIFU) is a completely non-invasive procedure that is being considered as an option with some dependence on tumor depth and anatomical complexity [38].

In conclusion, cryoablation stands out as a safe, effective, and minimally invasive approach for managing DTs, particularly when surgery is contraindicated or has failed. With increasing evidence supporting its clinical utility, cryoablation is becoming a key component in the multidisciplinary management of desmoid tumors [19,20,34,35,36,37].

Recommendation:

- Percutaneous cryoablation can be considered a reasonable local treatment option for small or medium-sized progressive or symptomatic extra-abdominal DTs (II, B).

### 4.3. Radiation Therapy

The role of RT in DT remains controversial. It can be considered an alternative for patients with tumors that have progressed despite systemic treatment or surgery, who cannot tolerate these treatments, or who experience significant pain or symptoms. It can also be considered as a first-line approach for cases where surgery may be highly morbid or result in positive margins [1,45]. DTs appear to be radiosensitive, but it is important to note that tumor regression after RT may take several years to occur [46].

A retrospective review of 22 articles reported local control rates of 61% with surgery, 75% with surgery plus RT, and 78% with RT alone. These results were analyzed according to margin status (positive, negative, or unknown) and whether the disease was primary, recurrent, or of unknown status [47]. The findings suggest that RT, whether alone or combined with surgery, provides better control regardless of margin status or disease type. Before resorting to amputative or mutilating surgery that compromises function or aesthetics, RT alone or tumor-reducing surgery followed by RT represents a reasonable alternative [40,42,48,49,50]. The recommended dose is 50–56 Gy in 2 Gy fractions. Higher doses do not reduce recurrence rates but increase toxicity, including fibrosis, edema, pathological fractures, soft tissue necrosis, anesthesia/paresthesia, and even radiation-induced tumors. The median time to these events is approximately 33 months, except for radiation-induced tumors, which typically develop after 10 years. These complications are more frequent in patients under 30 years of age.

A meta-analysis of 1295 patients across 16 studies concluded that postoperative RT is particularly recommended in cases of R1 or R2 resections due to the higher risk of recurrence, especially in recurrent tumors [28].

It is important to note that while several studies support the effectiveness of RT in desmoid fibromatosis, most are retrospective and have small sample sizes. Large-scale prospective clinical trials are needed; however, the low incidence of this disease and the diversity of tumor locations make it challenging to obtain solid evidence with sufficient statistical power [40].

Recommendation:

- RT should be reserved for progressive, symptomatic, or inoperable tumors when systemic therapies are contraindicated or ineffective, weighing potential long-term toxicities, particularly in patients under 30 years (IV, B).
- RT may be considered as an option in unresectable DMs or after incomplete (R1/R2) resections, particularly in recurrent cases, given its potential to improve local control (III, C).

### 4.4. Active Surveillance

Nowadays, most experts recommend AS as one of the initial management strategies for DT, based on spontaneous regression rates of approximately 20% reported in clinical studies [2,51]. This approach is indicated in asymptomatic patients or those with minimal symptoms who have tumors in non-critical locations. AS involves clinical and radiological follow-up (CT or MRI) every 8–12 weeks, at least during the initial phase of the disease, as most disease progression occurs within the first two years after diagnosis. In cases of progression, if the patient remains clinically stable, extending observation for an additional three months is recommended. If progression is confirmed, systemic therapy should be considered as the first-line intervention, excluding abdominal wall desmoids and highly symptomatic tumors in critical anatomical locations, where surgery remains the preferred treatment [23].

Recommendation:

- AS is recommended as the initial approach for asymptomatic or stable patients, given the potential for spontaneous stabilization or regression (III, A).

### 4.5. Systemic Therapy

The landscape of systemic treatment in DT has evolved significantly in recent years. Hormonal therapies (e.g., tamoxifen), with or without non-steroidal anti-inflammatory drugs (NSAIDs), are no longer recommended due to insufficient evidence of efficacy. In contrast, tyrosine kinase inhibitors (TKIs) and gamma-secretase inhibitors (GSIs) have shown greater efficacy and, in most cases, have replaced standard chemotherapy. Conventional chemotherapy—using anthracycline-based regimens, pegylated liposomal doxorubicin (preferred), or methotrexate with vinorelbine/vinblastine—is now generally reserved for the most aggressive cases. Doxorubicin, in conventional or liposomal form, has been used alone or with dacarbazine in small retrospective studies. No trials have compared it to newer agents. Anthracyclines show higher and faster response rates than tyrosine kinase inhibitors in clinical trials [52,53]. Methotrexate plus vinblastine is generally well-tolerated and effective, with responses ranging from minor reduction to complete remission over long follow-up. Newer oral agents are now preferred due to easier use and better tolerability [54].

TKIs have shown effectiveness in advanced and refractory desmoid tumors. Agents such as pazopanib (800 mg orally daily) [55] or imatinib (200–800 mg orally daily) [56] have demonstrated positive outcomes in phase II trials, though they are now considered secondary to sorafenib, which has shown superior efficacy. In a randomized phase III trial, sorafenib (400 mg orally daily) significantly reduced the risk of disease progression compared with placebo (HR 0.13; 95% CI, 0.05–0.31), with a 2-year progression free survival of 81% vs. 36% and overall response rate of 33% vs. 20%, respectively, with a manageable toxicity profile [57].

GSIs, which selectively inhibit the terminal portion of the NOTCH signaling pathway (intracellular domain), have demonstrated highly favorable results compared to placebo in a recently published phase III trial [58]. The treatment arm showed a 71% reduction in the risk of disease progression or death (HR = 0.29; 95% CI, 0.15–0.55; 0.001). Nirogacestat (150 mg orally twice daily), the first drug approved for this indication, received FDA approval in the United States in November 2023 for the treatment of adults with progressive DTs. This therapy demonstrated significant improvements in symptom control (pain, symptom severity, and quality of life) and presented a manageable toxicity profile compared to other available treatments. However, ovarian dysfunction in women of reproductive age has been frequently observed in approximately 70–75% of treated patients and is generally reversible upon treatment discontinuation. Other GSIs and compounds targeting the Wnt pathway are currently under investigation, with promising results anticipated that could expand the current therapeutic arsenal. Wnt-targeted therapies represent a novel approach in DTs, aiming to disrupt β-catenin–driven transcriptional activity central to tumorigenesis. Tegavivint (BC2059), a selective transducin β-like protein 1 (TBL1) inhibitor, promotes degradation of nuclear β-catenin and has shown preclinical efficacy in CTNNB1-mutant desmoid models [59]. In a phase 1 study, tegavivint was well tolerated and achieved an overall response rate of 17%, increasing to 25% at the recommended phase 2 dose, with all responses ongoing at a median duration of 8.1 months; it is currently being evaluated in a phase 2 trial (NCT03459469). Additional investigational strategies include small-molecule inhibitors acting upstream in the Wnt pathway and next-generation γ-secretase inhibitors, such as AL102 (RINGSIDE trial, NCT04871282), which target Notch–Wnt pathway crosstalk and have shown encouraging early efficacy and a favorable safety profile [60].

An overview of currently available drugs is provided in Table 2.

**Figure 4:**
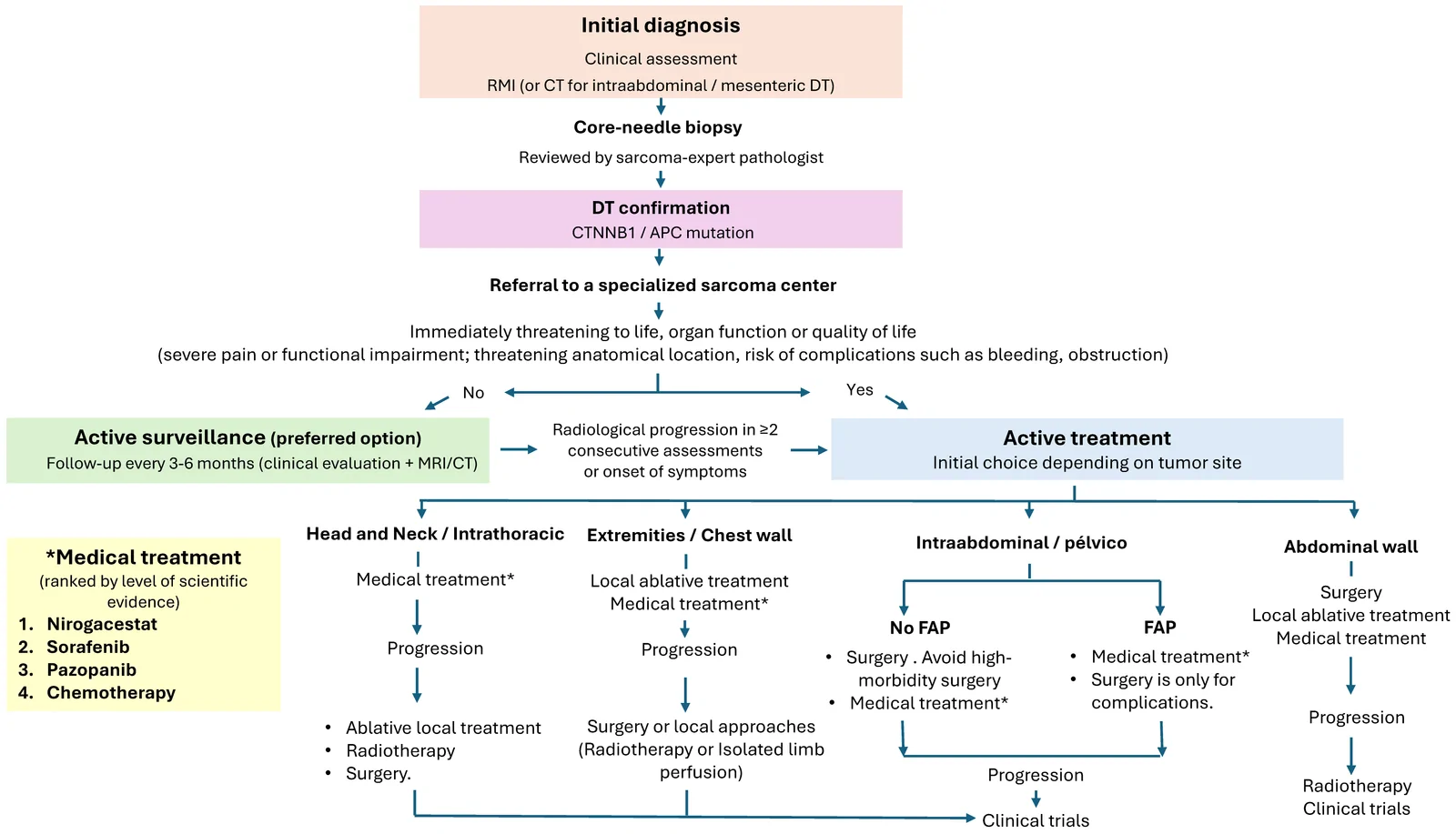
Algorithm for the Diagnosis and Management of DTs in Adult Patients.

The absence of head-to-head comparative studies makes it challenging to propose a definitive treatment sequence for systemic therapies in DTs. Therapeutic selection should be guided by the safety profiles of each agent in the context of individual patient characteristics. Generally, it is advisable to begin with agents that offer the best tolerability, escalating to more aggressive therapies as needed. At present, there are no robust data that enable us to determine the optimal duration of systemic therapy in DTs; however, a treatment period of at least six months is typically required before efficacy can be adequately evaluated. A proposed algorithm for the management of DTs in adults is shown in Figure 4.

Recommendations:

- Systemic treatment is indicated for symptomatic patients, rapid tumor progression, anatomical risk, or refractory or recurrent disease (III, A).
- Systemic Treatments for DTs:
  ○ Sorafenib is strongly recommended, supported by a randomized phase III trial (I, A);
  ○ Nirogacestat is strongly recommended following the results of the phase III DeFi trial (I, A);
  ○ Pazopanib may be considered in refractory or progressive cases (II, B);
  ○ Imatinib is also conditionally recommended in refractory disease. Its efficacy is variable, and evidence is primarily from small, non-randomized series (III, B);
  ○ Hormonal therapies, such as tamoxifen, are not recommended due to very-low-quality evidence and the lack of a proven benefit in modern clinical studies (IV, D);
  ○ Doxorubicin, either conventional or liposomal, with or without dacarbazine, is conditionally recommended for patients requiring rapid tumor control or with refractory/aggressive disease (III, B);
  ○ Methotrexate combined with vinblastine or vinorelbine is recommended as a first-line systemic therapy in pediatric and young adult patients (II, B).
- It is recommended to continue systemic therapies for at least 6 to 12 months before evaluating their effectiveness (IV, B).
- Inclusion in clinical trials for advanced disease patients is highly recommended (V, A).

## 5. Childhood Desmoid Tumors

The results of the Non-Rhabdomyosarcoma Soft Tissue Sarcomas trial 2005 from the European Pediatric Soft Tissue Sarcoma Group (NRSTS EpSSG 2005) confirm the current initial recommendation in childhood of the AS strategy, particularly for tumors in non-risk locations [61]. The tumor’s growth rate or potential spontaneous regression should be closely monitored. In cases of evident tumor progression, increasing pain, symptom worsening, or tumors in high-risk locations, initiating active treatment should be considered.

When treatment is necessary, the first-line recommendation is systemic therapy with minimal morbidity, such as the combination of low-dose intravenous methotrexate and vinblastine administered weekly for six months. After this period, the same doses are continued for another six months but administered every two weeks [62]. Reported chemotherapy outcomes include response rates of 35% for complete or partial responses (57% with VBL-MTX), with a further 45% of patients achieving stable disease. Tumor size greater than 5 cm has been identified as a prognostic factor [51]. Once stabilization is achieved, the role of surgery remains questionable, and continued observation is generally preferred to avoid the stimulation of tumor cells by the release of local growth factors during postoperative wound healing.

The clinical data of 173 patients with desmoid tumors included in the NRSTS EpSSG 2005 study between 2005 and 2016 were published. In 35% of cases, a wait-and-see approach was taken; in 31%, immediate surgery was performed; and in 34%, immediate chemotherapy was administered. The 5-year progression-free survival rate was 36.5% and did not differ between the three groups. Only one patient died from a secondary tumor [51].

In cases where first-line therapy is ineffective or disease progression continues, second-line options should be considered, with response rates ranging from 25% to 40%.

Available second-line treatment options for refractory DT in children comprise doxorubicin [63], alkaloid-based regimens [61], and hydroxyurea [64]. Based on the lack of efficacy demonstrated in adults, hormone therapy—with or without NSAIDs—should no longer be recommended in children.

Medical treatments include TKIs such as sorafenib or pazopanib, which are considered the first choice in most adult patients. The rationale for TKI use in pediatric DT is supported by their tolerable safety profile in other pediatric malignancies and by the need for alternatives to cytotoxic chemotherapy. However, the efficacy and long-term safety of TKIs in children with DT remain to be established in prospective trials, particularly concerning growth and fertility [65]. A recent national multicenter retrospective study of 50 pediatric patients with refractory DT after first-line therapy reported that second-line regimens were predominantly alkaloid-based, while TKIs were rarely incorporated due to limited pediatric access and evidence. The ORR to any medical therapy was 30%, with a 5-year PFS of 44%; however, specific outcomes for TKI-treated patients were not separately reported [66]. Systematic reviews confirm that most clinical trials of TKIs in DT have focused on adults, and pediatric inclusion remains rare, limiting the generalizability of adult data to children [67]. In summary, while TKIs are a promising option for pediatric DTF refractory to standard therapy, their incorporation into pediatric protocols awaits prospective, pediatric-specific trials to clarify efficacy, optimal sequencing, and long-term safety [66].

Due to the functional or aesthetic consequences that RT can have in childhood and the risk of developing long-term radiation-induced tumors, it only plays a role when several systemic therapies and surgeries have failed. Recently, cryotherapy has proven to be a safe and effective treatment modality for extra-abdominal desmoid tumors [68].

**Figure 5:**
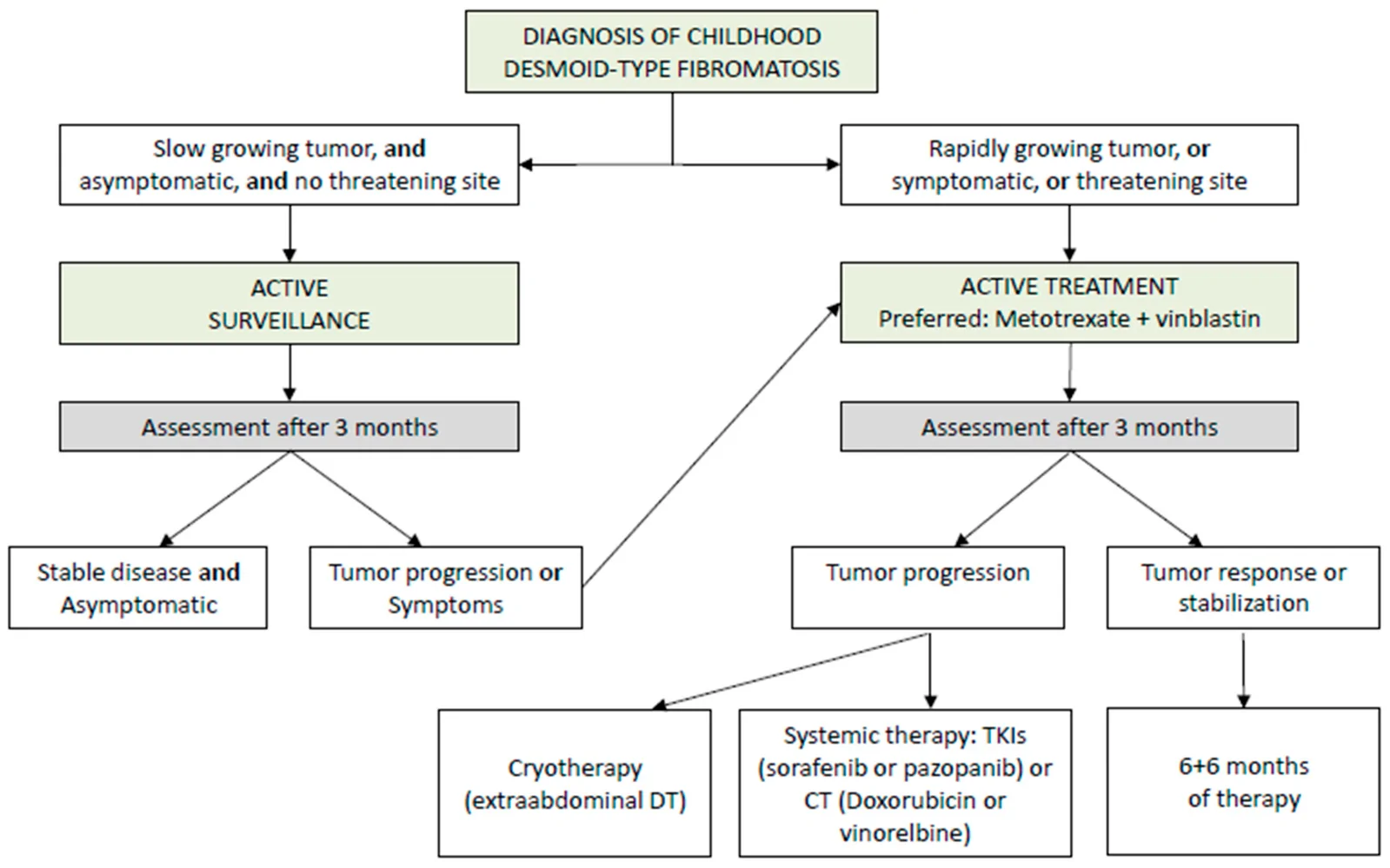
Flow chart for management of DT in children. Modified from NRSTS EpSSG 2005.

Recommendations:

- Initial management should employ AS for tumors in non-critical sites and without significant symptoms (V, B).
- Active treatment should be considered in cases of clear progression, increasing pain, worsening symptoms, or tumors in high-risk locations (V, B).
- When treatment is required, a multidisciplinary approach in reference centers is recommended, prioritizing non-mutilating strategies and avoiding upfront aggressive surgery (V, C) Figure 5.

## 6. Conclusions

DT is a rare disease that requires specialized treatment in high-volume referral centers with multidisciplinary teams. Thanks to recent advances in medical management, TKIs and GSIs, and new local treatments such as cryotherapy, patients now have a wide range of therapeutic options available. Given that DT is a localized neoplasm with no potential for metastasis and high survival rates, it is important to establish an appropriate management strategy for each patient, optimizing tumor control without compromising quality of life. Clear and pragmatic recommendations are essential to guide diagnosis and treatment and to optimize outcomes for patients with DTs.

## Figures and Tables

**Table 1:** Levels of evidence (I to V) and grades of recommendation (A to C).

Levels of Evidence	
I	Evidence from at least one large, randomized, controlled trial of good methodological quality (low potential for bias), or meta-analyses of well-conducted randomized trials without heterogeneity
II	Small randomized trials or large randomized trials with a suspicion of bias (lower methodological quality), or meta-analyses of such trials or of trials with demonstrated heterogeneity
III	Prospective cohort studies
IV	Retrospective cohort studies or case–control studies
V	Studies without a control group, case reports, and experts’ opinions
Grades of recommendation	
A	Strong evidence for efficacy with a substantial clinical benefit, strongly recommended
B	Strong or moderate evidence for efficacy but with a limited clinical benefit, generally recommended
C	Insufficient evidence for efficacy or benefit does not outweigh the risk or the disadvantages (adverse events, costs…), optional

**Table 2:** Systemic therapies for DTs.

Drug	Study Type (Key Trial)	N	Dose and Administration	ORR	PFS/Main Outcome	Toxicity
Conventional chemotherapy [52,53,54]	- Retrospective multicenter: MTX-VBL, - Anthracyclines - Phase II nab-paclitaxel	51 (MTS/VBL) 62 (Anthracyclines) 40	- MTX 30 mg/m^2^ + VBL 6 mg/m^2^ IV weekly or biweekly; - Anthracyclines 60–75 mg/m^2^ IV q3wk - Nab-paclitaxel 100–125 mg/m^2^ IV weekly	- MTX + VBL: PR 51%, CBR 95% - Anthracyclines 37–54% - Nab-paclitaxel 20%	- 5-yr PFS 80.8% (MTX + VBL) - year PFS 73% anthracyclines - 1-year PFS 91% nab-paclitaxel	- MTX + VBL: mild myelosuppression, neuropathy; - Anthracyclines/PLD: mucositis, cardiotoxicity, myelosuppression, nausea, fatigue, rash, etc.
Sorafenib [57] (TKI)	Phase III randomized, double-blind	50 sorafenib 37 placebo	- 400 mg orally once daily - Median time to response 9.6 mo	33% vs. 20% (placebo)	2-yr PFS 81% vs. 36%; HR 0.13 (0.05–0.31)	Rash, fatigue, hypertension, diarrhea (mostly G1–2)
Pazopanib [55] (TKI)	Phase II randomized non-comparative	48 pazopanib 24 MTS-VBL	800 mg orally once daily	37% PR (PZ) vs. 25% PR (MTX/VBL)	6-mo non-progression 83.7% (PZ) vs. 45.0% (MTX/VBL)	Pazopanib G3–4 toxicity: Hypertension (21%), diarrhea (15%)
Imatinib [56] (TKI)	Phase II multicenter. Retrospective series	51/40	400–800 mg orally once daily	6–19% (phase II); up to 23% (retrospective)	1-year PFS: 66–80%; 2-yr PFS 45–55%	Edema, fatigue, nausea, rash, mild cytopenias
Nirogacestat [58] (GSI)	Phase III randomized, double-blind.	70 nirogacestat 72 placebo	150 mg orally twice daily.	~41% vs. 8% (placebo)	HR 0.29 (0.15–0.55); Estimated 2-yr PFS 76% vs. 44% Significant improvement in pain and function	Diarrhea, nausea, fatigue, rash, hypophosphatemia; ovarian dysfunction (~75%, reversible in ~74%)

## References

[1] Penel N., Coindre J.M., Bonvalot S., Italiano A., Neuville A., Le Cesne A., Terrier P., Ray-Coquard I., Ranchere-Vince D., Robin Y.M. (2016). Management of desmoid tumours: A nationwide survey of labelled reference centre networks in France. Eur. J. Cancer.

[2] Fletcher C., Bridge J.A., Hogendoorn P.C.W., Mertens F., World Health Organization WHO (2013). WHO Classification of Tumours of Soft Tissue and Bone: WHO Classification of Tumours.

[3] Kasper B., Ströbel P., Hohenberger P. (2011). Desmoid Tumors: Clinical Features and Treatment Options for Advanced Disease. Oncologist.

[4] Kotiligam D., Lazar A.J., Pollock R.E., Lev D. (2008). Desmoid tumor: A disease opportune for molecular insights. Histol. Histopathol..

[5] Nieuwenhuis M.H.M., Lefevre J.H., Bülow S.M., Järvinen H.M., Bertario L.M., Kernéis S., Parc Y.M., Vasen H.F.A.M. (2011). Family history, surgery, and APC mutation are risk factors for desmoid tumors in familial adenomatous polyposis: An international cohort study. Dis. Colon Rectum.

[6] Santti K., Ihalainen H., Rönty M., Karlsson C., Haglund C., Sampo M., Tarkkanen M., Blomqvist C. (2019). Estrogen receptor beta expression correlates with proliferation in desmoid tumors. J. Surg. Oncol..

[7] Fiore M., Coppola S., Cannell A.J., Colombo C., Bertagnolli M.M., George S., Le Cesne A., Gladdy R.A., Casali P.G., Swallow C.J. (2014). Desmoid-type fibromatosis and pregnancy: A multi-institutional analysis of recurrence and obstetric risk. Ann. Surg..

[8] Cates J.M. (2015). Pregnancy does not increase the local recurrence rate after surgical resection of desmoid-type fibromatosis. Int. J. Clin. Oncol..

[9] Quintini C., Ward G., Shatnawei A., Xhaja X., Hashimoto K., Steiger E., Hammel J., Uso T.D., Burke C.A., Church J.M.M. (2012). Mortality of intra-abdominal desmoid tumors in patients with familial adenomatous polyposis: A single center review of 154 patients. Ann. Surg..

[10] National Comprehensive Cancer Network (NCCN) (2025). Soft Tissue Sarcoma. Version 5.2024. Plymouth Meeting (PA): NCCN. https://www.nccn.org/professionals/physician_gls/pdf/sarcoma.pdf.

[11] Van Houdt W.J., Wei I.H., Kuk D., Qin L.X., Jadeja B., Villano A., Hameed M., Singer S., Crago A.M. (2019). Yield of Colonoscopy in Identification of Newly Diagnosed Desmoid-Type Fibromatosis with Underlying Familial Adenomatous Polyposis. Ann. Surg. Oncol..

[12] Dykewicz C.A. (2001). Centers for Disease C, and Prevention. Infectious Diseases Society of A. American Society of B, Marrow T Summary of the guidelines for preventing opportunistic infections among hematopoietic stem cell transplant recipients. Clin. Infect. Dis..

[13] Rosa F., Martinetti C., Piscopo F., Buccicardi D., Schettini D., Neumaier C.E., Gandolfo N., Grazioli L., Gastaldo A. (2020). Multimodality imaging features of desmoid tumors: A head-to-toe spectrum. Insights Imaging.

[14] Braschi-Amirfarzan M., Keraliya A.R., Krajewski K.M., Tirumani S.H., Shinagare A.B., Hornick J.L., Baldini E.H., George S., Ramaiya N.H., Jagannathan J.P. (2016). Role of Imaging in Management of Desmoid-type Fibromatosis: A Primer for Radiologists. Radiographics.

[15] Faria S.C., Iyer R.B., Rashid A., Ellis L., Whitman G.J. (2004). Desmoid tumor of the small bowel and the mesentery. AJR Am. J. Roentgenol..

[16] Subhawong T.K., Feister K., Sweet K., Alperin N., Kwon D., Rosenberg A., Trent J., Wilky B.A. (2021). MRI Volumetrics and Image Texture Analysis in Assessing Systemic Treatment Response in Extra-Abdominal Desmoid Fibromatosis. Radiol. Imaging Cancer.

[17] Dinauer P.A., Brixey C.J., Moncur J.T., Fanburg-Smith J.C., Murphey M.D. (2007). Pathologic and MR imaging features of benign fibrous soft-tissue tumors in adults. Radiographics.

[18] Shinagare A.B., Ramaiya N.H., Jagannathan J.P., Krajewski K.M., Giardino A.A., Butrynski J.E., Raut C.P. (2011). A to Z of desmoid tumors. AJR Am. J. Roentgenol..

[19] Lee S.B., Oh S.N., Choi M.H., Rha S.E., Jung S.E., Byun J.Y. (2017). The imaging features of desmoid tumors: The usefulness of diffusion weighted imaging to differentiate between desmoid and malignant soft tissue tumors. Investig. Magn. Reson. Imaging.

[20] Kurtz J.E., Buy X., Deschamps F., Sauleau E., Bouhamama A., Toulmonde M., Honoré C., Bertucci F., Brahmi M., Chevreau C. (2021). CRYODESMO-O1: A prospective, open phase II study of cryoablation in desmoid tumour patients progressing after medical treatment. Eur. J. Cancer.

[21] Goldberg D., Woodhead G., Hannallah J., Young S. (2023). Role of the Interventional Radiologist in the Treatment of Desmoid Tumors. Life.

[22] Foster C.R., Strauss M., Hornick J.L., Habeeb O. (2023). Desmoid Fibromatosis with TP53 Mutation and Striking Nuclear Pleomorphism. Int. J. Surg. Pathol..

[23] Carlson J.W., Fletcher C.D. (2007). Immunohistochemistry for beta-catenin in the differential diagnosis of spindle cell lesions: Analysis of a series and review of the literature. Histopathology.

[24] Gronchi A., Miah A.B., Dei Tos A., Abecassis N., Bajpai J., Bauer S., Biagini R., Bielack S., Blay J.Y., Bolle S. (2021). ESMO Guidelines Committee, EURACAN and GENTURIS. Soft tissue and visceral sarcomas: ESMO-EURACAN-GENTURIS Clinical Practice Guidelines for diagnosis, treatment and follow-up^☆^. Ann. Oncol..

[25] Kasper B., Baldini E.H., Bonvalot S., Callegaro D., Cardona K., Colombo C., Corradini N., Crago A.M., Tos A.P.D., Dileo P. (2024). Desmoid Tumor Working Group. Current Management of Desmoid Tumors: A Review. JAMA Oncol..

[26] Schut A.W., Timbergen M.J.M., van Broekhoven D.L.M., van Dalen T., van Houdt W.J., Bonenkamp J.J., Sleijfer S., Grunhagen D.J., Verhoef C.A. (2023). Nationwide Prospective Clinical Trial on Active Surveillance in Patients With Non-intraabdominal Desmoid-type Fibromatosis: The GRAFITI Trial. Ann. Surg..

[27] Nieuwenhuis M.H., Casparie M., Mathus-Vliegen L.M., Dekkers O.M., Hogendoorn P.C., Vasen H.F. (2011). A nation-wide study comparing sporadic and familial adenomatous polyposis-related desmoid-type fibromatoses. Int. J. Cancer.

[28] Crago A.M., Chmielecki J., Rosenberg M., O’Connor R., Byrne C., Wilder F.G., Thorn K., Agius P., Kuk D., Socci N.D. (2015). Near universal detection of alterations in CTNNB1 and Wnt pathway regulators in desmoid-type fibromatosis by whole-exome sequencing and genomic analysis. Genes Chromosomes Cancer.

[29] Janssen M.L., van Broekhoven D.L., Cates J.M., Bramer W.M., Nuyttens J.J., Gronchi A., Salas S., Bonvalot S., Grünhagen D.J., Verhoef C. (2017). Meta-analysis of the influence of surgical margin and adjuvant radiotherapy on local recurrence after resection of sporadic desmoid-type fibromatosis. Br. J. Surg..

[30] Canovai E., Butler A., Clark S., Latchford A., Sinha A., Sharkey L., Rutter C., Russell N., Upponi S., Amin I. (2024). Treatment of Complex Desmoid Tumors in Familial Adenomatous Polyposis Syndrome by Intestinal Transplantation. Transplant. Direct..

[31] Wilkinson M.J., Chan K.E., Hayes A.J., Strauss D.C. (2014). Surgical outcomes following resection for sporadic abdominal wall fibromatosis. Ann. Surg. Oncol..

[32] Catania G., Ruggeri L., Iuppa G., Di Stefano C., Cardi F., Iuppa A. (2012). Abdominal wall reconstruction with intraperitoneal prosthesis in desmoid tumors surgery. Updates Surg..

[33] Sutton R.J., Thomas J.M. (1999). Desmoid tumours of the anterior abdominal wall. Eur. J. Surg. Oncol..

[34] Bertani E., Chiappa A., Testori A., Mazzarol G., Biffi R., Martella S., Pace U., Soteldo J., Vigna P.D., Lembo R. (2009). Desmoid tumors of the anterior abdominal wall: Results from a monocentric surgical experience and review of the literature. Ann. Surg. Oncol..

[35] Bonvalot S., Ternès N., Fiore M., Bitsakou G., Colombo C., Honoré C., Marrari A., Le Cesne A., Perrone F., Dunant A. (2013). Spontaneous regression of primary abdominal wall desmoid tumors: More common than previously thought. Ann. Surg. Oncol..

[36] Kasper B., Baumgarten C., Bonvalot S., Haas R., Haller F., Hohenberger P., Moreau G., van der Graaf W., Gronchi A. (2015). Management of sporadic desmoid-type fibromatosis: A European consensus approach based on patients’ and professionals’ expertise—a sarcoma patients EuroNet and European Organisation for Research and Treatment of Cancer/Soft Tissue and Bone Sarcoma Group initiative. Eur. J. Cancer.

[37] Borghi A., Gronchi A. (2023). Desmoid tumours (extra-abdominal), a surgeon’s nightmare. Bone Jt. J..

[38] Fernandez M.M., Bell T., Tumminello B., Khan S., Zhou S., Oton A.B. (2023). Disease and economic burden of surgery in desmoid tumors: A review. Expert. Rev. Pharmacoecon Outcomes Res..

[39] Nishida Y., Hamada S., Kawai A., Kunisada T., Ogose A., Matsumoto Y., Ae K., Toguchida J., Ozaki T., Hirakawa A. (2020). Risk factors of local recurrence after surgery in extraabdominal desmoid-type fibromatosis: A multicenter study in Japan. Cancer Sci..

[40] Papalexis N., Savarese L.G., Peta G., Errani C., Tuzzato G., Spinnato P., Ponti F., Miceli M., Facchini G. (2023). The New Ice Age of Musculoskeletal Intervention: Role of Percutaneous Cryoablation in Bone and Soft Tissue Tumors. Curr. Oncol..

[41] Pal K., Awad A., Yevich S., Kuban J.D., Tam A.L., Huang S.Y., Odisio B.C., Gupta S., Habibollahi P., Bishop A.J. (2024). Safety and Efficacy of Percutaneous Cryoablation for Recurrent or Metastatic Soft-Tissue Sarcoma in Adult Patients. AJR Am. J. Roentgenol..

[42] Auloge P., Cazzato R.L., Rousseau C., Caudrelier J., Koch G., Rao P., Chiang J.B., Garnon J., Gangi A. (2019). Complications of Percutaneous Bone Tumor Cryoablation: A 10-year Experience. Radiology.

[43] Alaseem A., Alsaikhan N., AlSudairi A.M., Alsehibani Y.A., Alhuqbani M.N., Aldosari Z.A., Aldosari O.A., Almuhanna A., Alshaygy I.S. (2025). Systematic review of transarterial chemoembolization for desmoid tumors: A promising locoregional treatment for challenging tumor. Discov. Oncol..

[44] Chetan M., Gillies M., Rehman S., McCarthy C., Cosker T., Wu F., Lyon P.C. (2025). High-intensity focused ultrasound treatment of unresectable soft tissue sarcoma and desmoid tumours—A systematic review. Clin. Radiol..

[45] Ibrahim R., Assi T., Khoury R., Ngo C., Faron M., Verret B., Lévy A., Honoré C., Hénon C., Le Péchoux C. (2024). Desmoid-type fibromatosis: Current therapeutic strategies and future perspectives. Cancer Treat. Rev..

[46] Matsunobu T., Kunisada T., Ozaki T., Iwamoto Y., Yoshida M., Nishida Y. (2020). Definitive radiation therapy in patients with unresectable desmoid tumors: A systematic review. Jpn. J. Clin. Oncol..

[47] Nuyttens J.J., Rust P.F., Thomas C.R., Turrisi A.T. (2000). Surgery versus radiation therapy for patients with aggressive fibromatosis or desmoid tumors: A comparative review of 22 articles. Cancer.

[48] Gronchi A., Colombo C., Le Péchoux C., Dei Tos A.P., Le Cesne A., Marrari A., Penel N., Grignani G., Blay J.Y., Casali P.G. (2014). Sporadic desmoid-type fibromatosis: A stepwise approach to a non-metastasising neoplasm--a position paper from the Italian and the French Sarcoma Group. Ann. Oncol..

[49] Guadagnolo B.A., Zagars G.K., Ballo M.T. (2008). Long-term outcomes for desmoid tumors treated with radiation therapy. Int. J. Radiat. Oncol. Biol. Phys..

[50] Keus R.B., Nout R.A., Blay J.Y., de Jong J.M., Hennig I., Saran F., Hartmann J.T., Sunyach M.P., Gwyther S.J., Ouali M. (2013). Results of a phase II pilot study of moderate dose radiotherapy for inoperable desmoid-type fibromatosis--an EORTC STBSG and ROG study (EORTC 62991-22998). Ann Oncol..

[51] Timbergen M.J.M., Schut A.W., Grünhagen D.J., Sleijfer S., Verhoef C. (2020). Active surveillance in desmoid-type fibromatosis: A systematic literature review. Eur. J. Cancer.

[52] De Camargo V.P., Keohan M.L., D’Adamo D.R., Antonescu C.R., Brennan M.F., Singer S., Ahn L.S., Maki R.G. (2010). Clinical outcomes of systemic therapy for patients with deep fibromatosis (desmoid tumor). Cancer.

[53] Constantinidou A., Jones R.L., Scurr M., Al-Muderis O., Judson I. (2009). Pegylated liposomal doxorubicin, an effective, well-tolerated treatment for refractory aggressive fibromatosis. Eur. J. Cancer.

[54] Azzarelli A., Gronchi A., Bertulli R., Tesoro J.D., Baratti D., Pennacchioli E., Dileo P., Rasponi A., Ferrari A., Pilotti S. (2001). Low-dose chemotherapy with methotrexate and vinblastine for patients with advanced aggressive fibromatosis. Cancer.

[55] Toulmonde M., Pulido M., Ray-Coquard I., Andre T., Isambert N., Chevreau C., Penel N., Bompas E., Saada E., Bertucci F. (2019). Pazopanib or methotrexate-vinblastine combination chemotherapy in adult patients with progressive desmoid tumours (DESMOPAZ): A non-comparative, randomised, open-label, multicentre, phase 2 study. Lancet Oncol..

[56] Kasper B., Gruenwald V., Reichardt P., Bauer S., Rauch G., Limprecht R., Sommer M., Dimitrakopoulou-Strauss A., Pilz L., Haller F. (2017). Imatinib induces sustained progression arrest in RECIST progressive desmoid tumours: Final results of a phase II study of the German Interdisciplinary Sarcoma Group (GISG). Eur. J. Cancer.

[57] Gounder M.M., Mahoney M.R., Van Tine B.A., Ravi V., Attia S., Deshpande H.A., Gupta A.A., Milhem M.M., Conry R.M., Movva S. (2018). Sorafenib for Advanced and Refractory Desmoid Tumors. N. Engl. J. Med..

[58] Gounder M., Ratan R., Alcindor T., Schöffski P., van der Graaf W.T., Wilky B.A., Riedel R.F., Lim A., Smith L.M., Moody S. (2023). Nirogacestat, a γ-Secretase Inhibitor for Desmoid Tumors. N. Engl. J. Med..

[59] Braggio D.A., Costas Cde Faria F., Koller D., Jin F., Zewdu A., Lopez G., Batte K., Casadei L., Welliver M., Horrigan S.K. (2022). Preclinical efficacy of the Wnt/β-catenin pathway inhibitor BC2059 for the treatment of desmoid tumors. PLoS ONE.

[60] Gounder M., Jones R.L., Chugh R., Agulnik M., Singh A.S., Van Tine B.A., Andelkovic V., Choy E., Lewing J.H., Ratan R. (2023). RINGSIDE phase 2/3 trial of AL102 for treatment of desmoid tumors (DT): Phase 2 results. J. Clin. Oncol..

[61] Orbach D., Brennan B., Bisogno G., Van Noesel M., Minard-Colin V., Daragjati J., Casanova M., Corradini N., Zanetti I., De Salvo G.L. (2017). The EpSSG NRSTS 2005 treatment protocol for desmoid-type fibromatosis in children: An international prospective case series. Lancet Child Adolesc. Health.

[62] Ferrari A., Brennan B., Casanova M., Corradini N., Berlanga P., Schoot R.A., Ramirez-Villar G.L., Safwat A., Guillen Burrieza G., Dall’Igna P. (2022). Pediatric Non-Rhabdomyosarcoma Soft Tissue Sarcomas: Standard of Care and Treatment Recommendations from the European Paediatric Soft Tissue Sarcoma Study Group (EpSSG). Cancer Manag. Res..

[63] Constantinidou A., Jones R.L., Scurr M., Judson I. (2011). Advanced aggressive fibromatosis: Effective palliation with chemotherapy. Acta Oncol..

[64] Ferrari A., Orbach D., Affinita M.C., Chiaravalli S., Corradini N., Meazza C., Bisogno G., Casanova M. (2019). Evidence of hydroxyurea activity in children with pretreated desmoid-type fibromatosis: A new option in the armamentarium of systemic therapies. Pediatr. Blood Cancer.

[65] Sparber-Sauer M., Orbach D., Navid F., Hettmer S., Skapek S., Corradini N., Casanova M., Weiss A., Schwab M., Ferrari A. (2021). Rationale for the use of tyrosine kinase inhibitors in the treatment of paediatric desmoid-type fibromatosis. Br. J. Cancer.

[66] Borovkov A., Orbach D., Bonneau-Lagacherie J., Carausu L., Guimard G., Haouy S., Jannier S., Pagnier A., Proust S., Verite C. (2025). Desmoid-TypeFibromatosis Tumors in Children After First-Line Failure: Clinical Aspects and Approaches for Subsequent Therapeutic Lines. Pediatr. Blood Cancer.

[67] van Maren S.A., van Noesel M.M., Husson O., van der Graaf W.T.A. (2022). Clinical trials in desmoid-type fibromatosis in children and adults: A systematic review. Pediatr. Blood Cancer.

[68] Vora B.M.K., Munk P.L., Somasundaram N., Ouellette H.A., Mallinson P.I., Sheikh A., Abdul Kadir H., Tan T.J., Yan Y.Y. (2021). Cryotherapy in extra-abdominal desmoid tumors: A systematic review and meta-analysis. PLoS ONE.

